# Conceptualising the interactive effects of climate change and biological invasions on subarctic freshwater fish

**DOI:** 10.1002/ece3.2982

**Published:** 2017-04-26

**Authors:** Robert J. Rolls, Brian Hayden, Kimmo K. Kahilainen

**Affiliations:** ^1^ Institute for Applied EcologyUniversity of CanberraCanberraACTAustralia; ^2^ Kilpisjärvi Biological StationUniversity of HelsinkiKilpisjärviFinland; ^3^ Department of Environmental SciencesUniversity of HelsinkiHelsinkiFinland; ^4^ Biology DepartmentCanadian Rivers InstituteUniversity of New BrunswickFrederictonNBCanada

**Keywords:** community interaction, extreme climatic events, food web, invasive species, range expansion

## Abstract

Climate change and species invasions represent key threats to global biodiversity. Subarctic freshwaters are sentinels for understanding both stressors because the effects of climate change are disproportionately strong at high latitudes and invasion of temperate species is prevalent. Here, we summarize the environmental effects of climate change and illustrate the ecological responses of freshwater fishes to these effects, spanning individual, population, community and ecosystem levels. Climate change is modifying hydrological cycles across atmospheric, terrestrial and aquatic components of subarctic ecosystems, causing increases in ambient water temperature and nutrient availability. These changes affect the individual behavior, habitat use, growth and metabolism, alter population spawning and recruitment dynamics, leading to changes in species abundance and distribution, modify food web structure, trophic interactions and energy flow within communities and change the sources, quantity and quality of energy and nutrients in ecosystems. Increases in temperature and its variability in aquatic environments underpin many ecological responses; however, altered hydrological regimes, increasing nutrient inputs and shortened ice cover are also important drivers of climate change effects and likely contribute to context‐dependent responses. Species invasions are a complex aspect of the ecology of climate change because the phenomena of invasion are both an effect and a driver of the ecological consequences of climate change. Using subarctic freshwaters as an example, we illustrate how climate change can alter three distinct aspects of species invasions: (1) the vulnerability of ecosystems to be invaded, (2) the potential for species to spread and invade new habitats, and (3) the subsequent ecological effects of invaders. We identify three fundamental knowledge gaps focused on the need to determine (1) how environmental and landscape characteristics influence the ecological impact of climate change, (2) the separate and combined effects of climate and non‐native invading species and (3) the underlying ecological processes or mechanisms responsible for changes in patterns of biodiversity.

## 1 Introduction

Understanding and predicting the consequences of climate change and extreme climate events on global biodiversity has become a core research theme in ecology (Diez et al., 2012; Grimm et al., 2013; Jentsch, Kreyling, & Beierkuhnlein, 2007; Thompson, Beardall, Beringer, Grace, & Sardina, 2013; Thuiller, 2007). Consequently, there is a strong interest in identifying general, global principles of climate change impacts *among* biomes, ecosystem types, and levels of ecological organization (Harley et al., 2006; Harrod, 2015; Scheffers et al., 2016; Woodward, Perkins, & Brown, 2010). However, the search for general principles requires understanding of the ecological effects of climate change *within* biomes and ecosystem types to determine context‐specific responses. In this synthesis, we illustrate the effects of climate change on freshwater fish across levels of ecological organization (individual, population, community, and ecosystem) and highlight how effects of climate change are driven by physical environmental change, effects of invasive species, and their interactions. Our focus is on high latitude subarctic freshwater ecosystems (taiga or tundra ecosystems characterized by long, cold winters and short, cool summers; Nilsson, Polvi, & Lind, 2015), described as “sentinel systems” of the effects of climate change owing to their pronounced changes relative to other regions worldwide and location expected to meet significant range expansion of freshwater species (Heino, Virkkala, & Toivonen, 2009; Nilsson et al., 2015; Perkins, Reiss, Yvon‐Durocher, & Woodward, 2010; Wrona, Prowse, Reist, & Hobbie, 2006).

Climate change is most widely recognized for its effect on altered species distribution due to changes in thermal regimes (Buisson, Grenouillet, Villéger, Canal, & Laffaille, 2013; Parmesan & Yohe, 2003; Walther et al., 2002), emphasizing that species invasions are a potential mechanism driving climate change impacts. Here, we define invasive species as those that are expanding their distribution in response to climate change separate from species that have been moved by human agents beyond their natural range (Rahel & Olden, 2008; Walther et al., 2009). For freshwater biota, particularly fish, this is evidenced by shifts in the distribution of warm‐ or cool‐water species toward higher elevations and latitudes and range retractions for cold‐water species (Bond, Thomson, Reich, & Stein, 2011; Chu, Mandrak, & Minns, 2005; Comte, Buisson, Daufresne, & Grenouillet, 2013; Graham & Harrod, 2009). Ecological effects of climate change therefore need to be understood in the context of multiple stressors because the effects of species invasions and climate change may be synergistic or antagonistic (sensu Folt, Chen, Moore, & Burnaford, 1999; Strayer, 2010). However, the ecological effects of climate change and species invasions are often explored in isolation or with little consideration of the effects of other environmental disturbances (e.g., Cucherousset & Olden, 2011; Rehage & Blanchard, 2016; Simon & Townsend, 2003; Sousa, Novais, Costa, & Strayer, 2014; Woodward et al., 2010). Current conceptualizations of invasive species under climate change research emphasize how climate change may potentially alter invasion opportunities and routes (Rehage & Blanchard, 2016), with only a weaker understanding of how climate change determines the effects of invaders (e.g., Rahel & Olden, 2008, who propose how climate change can enhance or negate effects of freshwater invasive species). However, there has been increasing primary research effort in the processes and effects of invasive species under climate change in subarctic freshwaters since these initial theoretical developments. Therefore, there remains a dual need to first conceptualize how ecological effects of climate change are driven indirectly by the impacts of invasive species and second, how the effects of climate change influence the vulnerability of ecosystems to invasion and subsequent ecological responses.

Poleward range expansions of temperate freshwater species (e.g., Comte et al., 2013) further signify the importance of subarctic regions as sentinel ecosystems for understanding climate change impacts. Previous reviews of the consequences of climate change for freshwater ecosystems (including those including subarctic regions) have emphasized (1) the responses of hydrological and physical environmental components (Prowse et al., 2006), (2) effects on species range shifts and ecosystem function (Heino et al., 2009; Wrona et al., 2006), and (3) general responses across multiple levels of ecological organization in freshwaters predominantly independent of the effects of species invasions (Woodward et al., 2010). While our understanding of how patterns of freshwater biodiversity are expected to respond to climate change is developing (Comte et al., 2013; Heino et al., 2009), how the responses to climate are integrated among multiple levels of organization in subarctic freshwaters have not yet been conceptualized. Such a synthesis is useful to both predict the context‐dependent responses to environmental or ecological changes and identify underlying causal mechanisms for future research (Simon & Townsend, 2003).

Identifying the mechanistic linkages between environmental drivers and ecological responses is essential for predicting the effects of climate change, particularly to determine the extent to which generalizations can be made. The aim of this synthesis is to explore the effects of climate change across multiple levels of ecological organization in subarctic freshwaters focussing primarily on fish. We first present a summary of the recorded or predicted environmental outcomes of climate change, including the encroachment of temperate species into subarctic regions before then illustrating how these changes manifest as ecological responses at individual, population, community, and ecosystem levels of organization. Changes in species distributions are a ubiquitous consequence of climate change and concern for conservation management. In the final section, we explore the invasion triangle concept (Perkins, Leger, & Nowak, 2011) in subarctic freshwaters to provide an example of how the connections between climate change and species invasions need to be considered simultaneously, occasionally drawing on additional evidence from temperate regions.

## 2 Effects of Climate Change on Subarctic Freshwaters: Drivers of Potential Ecological Consequences

**Figure 1:**
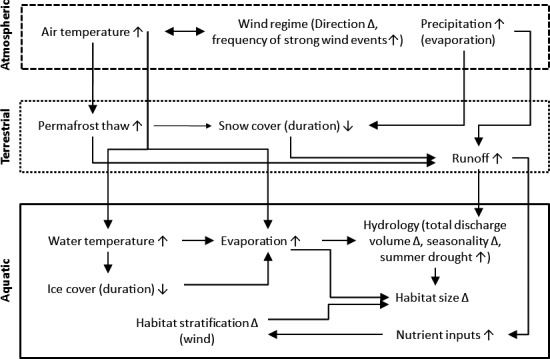
Schematic diagram representing the relationships between atmospheric, terrestrial, and aquatic components of subarctic and arctic freshwaters, and the processes by which climate change alters these environments. Linkages, specifically the effect of evaporation on precipitation, effect of wind regime on both ice‐cover duration and habitat stratification, are not shown to maintain clarity (see text for details). These linkages show that many of the environmental and ecological effects of climate change occur due to changes across all components of the subarctic environment. Arrows indicate the recorded or predicted direction of change (increases or decreases), and delta (∆) represents change that is not broadly generalizable within the region.

Interpreting and predicting the effects of climate change on freshwater ecosystems requires an understanding of how changes in climate alter specific physical and biological features relevant to their structure and function. Broadly, climate change alters the atmospheric, terrestrial, and aquatic components of freshwater ecosystems with linkages among each component (Figure 1; Table 1).

**Table 1:** Summary of the physical and chemical responses to climate change in northern freshwater environments

Variable	Key responses and examples	Sources
Temperature (air)	Global mean temperature has increased by 0.85°C during 1880–2010 Subarctic annual average temperature increases since 1980 have been have been twice that of the rest of the world If a global upper limit of 2°C mean temperature increase of human‐induced warming is set, the Arctic and Subarctic regions are expected to warm by 2.8–7.8°C	IPCC (2013) AMAP (2012) CAFF (2013)
	Subarctic mean temperature has increased by 1.2°C during 1948–2005 (northern Canada)	Prowse et al. (2009)
	Temperature increase in Siberia, Russia, has occurred at four times that of the global average during the 20th–21st centuries	Mokhov (2009)
Temperature (water)	Mean water temperature has increased by 0.3°C per decade in subarctic and alpine regions of North America during the 20th century	Chapin et al. (2000), Isaak et al. (2010)
	Increases in water temperatures vary significantly across season, with winter and spring experiencing greater increases than in summer and autumn (northern Europe)	Elliott & Elliott (2010)
	Small lakes and streams are expected to experience greater variation and changes when compared with larger systems due to reduced thermal buffering (whole subarctic)	Heino et al. (2009)
Ice cover and snow	Later lake‐ice formation and earlier ice thaw and breakup (e.g., in the Torne River bordering Finland and Sweden, the date of river ice breakup was 0.66 days earlier per decade between 1867 and 2013, compared to 0.30 days earlier per decade between 1693–1866)	Sharma et al. (2016)
	Increased duration of ice‐free periods (e.g., by up to 35 days between 2035 and 2070 predicted, when compared with 1960–1991) (whole subarctic)	Benson et al. (2012), Carter & Schindler (2012), Dibike et al. (2012), Godiksen et al. (2012), Magnuson et al. (2000)
Precipitation	Mean precipitation increased by 16%–25% between 1948 and 2004 in northern Canada	Prowse et al. (2009)
Hydrology	Increased annual discharge by 7% during the 20th century (rivers draining into the Arctic Ocean)	Peterson et al. (2002)
	Earlier Spring flooding due to ice thaw and snowmelt (subarctic USA)	Goode et al. (2013)
	Increased frequency and duration of low flow during summer; increased intra‐ and interannual hydrological variability (subarctic USA)	Davis, et al. (2013), Sinnatamby et al. (2012)
	Increased intra‐annual water level fluctuation in boreal lakes	Heino et al. (2009)
Wind	Possible increases in wind due to loss of Arctic sea ice, leading to reduced stratification in water bodies	Wu et al. (2013)
Chemical elements	Increased inputs of organic carbon due to increased runoff from terrestrial zones (subarctic Finland, Norway, USA)	Hobbie et al. (1999), Larsen et al. (2011), Räike, Kortelainen, Mattsson, & Thomas (2016)
	Increased release of phosphorous and nitrogen with greater thawing of permafrost (subarctic USA)	Jones, Booth, Yu, & Ferry (2013)
	Decreased dissolved oxygen in hypolimnetic zones of lakes due to stratification (subarctic Norway, USA)	Hobbie et al. (1999), Lindholm et al. (2012)
	Increased turbidity in water bodies due to the combined effects of increased temperature, precipitation, runoff, and changing wind regimes	Kernan et al. (2010)
Trophic state	Increased occurrence and duration of eutrophic conditions (subarctic)	Jeppesen et al. (2010)

### 2.1 Air and water temperature

**Figure 2:**
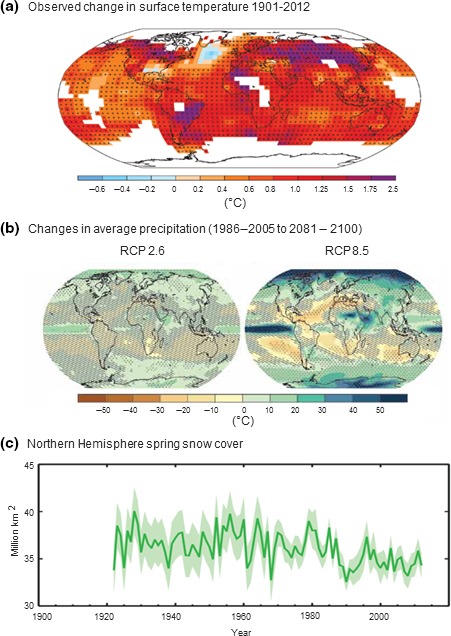
Selection of observed and modeled changes in climate variables over time assessed in the IPCC 5th Assessment Report (IPCC, 2013) (a) Map of observed surface temperature change from 1901 to 2012. Temperature trends determined by linear regression of combined land‐surface air and sea surface temperature datasets. Trends have been calculated where data availability permits a robust estimate (i.e., only for grid boxes with >70% complete records and more than 20% data availability in the first and last 10% of the time period). Other areas are white. Grid boxes where the trend is significant at the 10% level are indicated by a + sign. (b) Map of multimodel mean results for the IPCC Working Group 5 scenarios RCP2.6 (conservative warming) and RCP8.5 (extreme warming) in 2081–2100 of average percent change in annual mean precipitation relative to 1986–2005, the number of models used to calculate the multimodel mean is indicated in the upper right corner. (c) Extent of Northern Hemisphere March–April (spring) average snow cover, solid line indicates annual values, uncertainties are indicated by shading. Figures modified with permission from (a) Figure SPM.1, (b) Figure SPM.8, and (c) Figure SPM.3 in Summary for Policy Makers in IPCC (2013).

Global air surface temperatures have (on average) risen by 0.85°C since 1880, exceeding rates experienced in previous centuries (IPCC, 2013), and these increases are disproportionately higher in northern regions of the Earth (Figure 2a). For example, mean surface temperatures across Canada increased by 1.2°C during 1948–2005; however, these increases were greatest in western (2–2.2°C) arctic and subarctic regions (Prowse, Furgal, Bonsal, & Edwards, 2009). Recent temperature increases in Siberia (Russia) are four times greater than global averages (Mokhov, 2009). Predictions of future temperatures indicate that recent historical trends will persist in the remainder of the 21st century and that warming in the subarctic and arctic regions will also continue to exceed global averages (IPCC, 2013; Kjellström et al., 2013). Such predictions are consistent across studies despite variation between model outputs and estimates of future greenhouse gas emissions (Tebaldi, Arblaster, & Knutti, 2011).

As water temperature is primarily determined by ambient air temperature and solar radiation (Luoto & Nevalainen, 2013), increasing air temperature is reflected in the temperature of freshwaters. Mean annual water temperatures have increased by 0.3°C per decade during the 20th century in subarctic and arctic regions and comparable alpine streams in the USA (Chapin et al., 2000; Isaak et al., 2010). However, changes in mean annual water temperature are not consistent across all seasons. Water temperatures in winter and spring in northwestern Europe have increased by 0.4°C per decade, yet have not altered significantly in summer and autumn seasons (Elliott & Elliott, 2010). Physical environmental characteristics of lakes and rivers have a strong influence on thermal regimes, whereby large volumes of water (e.g., deep lakes) “dampen” temperature variation when compared to small and shallow habitats (e.g., small streams; Heino et al., 2009). In the absence of changes in runoff, increases in water temperature may decrease the size of habitats via evaporation, potentially resulting in complete disappearance of small lakes and seasonal drying of intermittent streams (Chen, Rowland, Wilson, Altmann, & Brumby, 2014; Wrona et al., 2013).

### 2.2 Precipitation and hydrological regimes

Increasing air and water temperatures are expected to alter regional weather patterns, particularly precipitation regimes in subarctic regions (Palmer & Räisänen, 2002). Mean total annual precipitation increased by 16%–25% during 1948–2004 across different climate regions of northern Canada (Prowse et al., 2009) and these increases are predicted to continue throughout the 21st century (IPCC, 2013; Figure 2b). However, the Arctic Oscillation and North Atlantic Oscillation are predicted to cause significant interannual variation in precipitation (Wu, Handorf, Dethloff, Rinke, & Hu, 2013). The proportion of precipitation as rainfall (in contrast to snow) is also expected to increase as extreme events, likely resulting in increased severity and “flashiness” of flooding (Nilsson et al., 2015).

Subarctic freshwaters experience two contrasting seasons: ice‐free summer and an ice‐covered winter (Hampton et al., 2017; McMeans, McCann, Humphries, Rooney, & Fisk, 2015). Increases in water temperature related to climate change are most apparent in terms of changes in the timing and duration of ice cover (e.g., Benson et al., 2012; Duguay et al., 2006; Korhonen, 2006; Lei, Leppäranta, Cheng, Heil, & Li, 2012; Sharma & Magnuson, 2014; Wang et al., 2012). Critically, while natural climate oscillations (e.g., North Atlantic Oscillation) affect the timing of ice thaw in Northern Hemisphere lakes, they do not conceal progressively earlier dates of thawing during 1855–2004 (Sharma & Magnusson, 2014). For example, lake ice‐cover duration decreased by 0.7–4.3 days per decade in lakes across the subarctic due to both later freeze and earlier ice breakup between 1855 and 2005 (Benson et al., 2012; Magnuson et al., 2000). Analyses of river ice breakup dates in the Torne River (bordering Finland and Sweden) during 1693–2013 determined a trend of earlier ice thaw by 0.66 days per decade during 1867–2013 (compared with 0.30 days per decade during 1693–1866) (Sharma et al., 2016). Such observations are expected to continue in the future, with predictions of reduced duration of winter ice cover by up to 35 days during 2035–2070 when compared with 1960–1991 (Dibike, Prowse, Bonsal, Rham, & Saloranta, 2012).

Discharge of subarctic rivers has increased and is predicted to rise further, with climate change. During the 20th century, total annual discharge increased by 7% among rivers of northern Eurasia (Peterson et al., 2002). Intra‐annual discharge seasonality is also shifting due to earlier thawing of ice and snow. Specifically, a shortening of the winter snow cover period by 30–50 days is predicted to bring the timing of annual flood peaks forward by approximately 20 days in catchments draining into the Arctic Ocean (Dankers & Middelkoop, 2008). Earlier spring thawing of snow and ice are expected to intensify low‐flow conditions and habitat contraction during summer, particularly for small and shallow rivers and lakes (Bouchard et al., 2013; Davis, et al., 2013; Goode et al., 2013; Sinnatamby, Babaluk, Power, Reist, & Power, 2012). However, the severity of flow reductions during summer periods also depends on the effects of climate change on seasonal precipitation regimes. For example, rainfall and extreme rainfall events are predicted to shift toward winter periods (Nilsson et al., 2015), further contributing to seasonal contraction of aquatic environments.

### 2.3 Wind regimes

Climate change is altering wind regimes in the subarctic and arctic zones, specifically the frequency of extreme wind events and direction. In the context of climate change, wind regimes in the subarctic and arctic are influenced by the extent of sea ice in the Arctic Ocean (Wu et al., 2013). Since 1979, there has been a fourfold increase in the number of high wind events in northern Alaska, USA, associated with the extent and volume of Arctic sea ice influencing prevailing weather conditions (Hinzman et al., 2005; Wu et al., 2013). Wind is relevant to freshwater environments, particularly standing water bodies. Oxygen and temperature stratification during summer periods limits the productive layer of subarctic lakes, and this stratification can be eliminated by wind turbulence (Hinzman et al., 2005). Further increases in the frequency of extreme wind events are predicted (Wu et al., 2013), potentially altering ecosystem productivity in lakes.

### 2.4 Nutrient inputs and productivity

Changing climate in the subarctic is expected to increase the transport and availability of elements and basal nutrients via greater runoff into freshwaters from terrestrial zones, which has a major effect on ecosystem productivity. Dissolved organic carbon (DOC) is an important source of energy for clear water ecosystems (e.g., Pace et al., 2007) and the availability of both DOC and phosphorous control primary production in subarctic freshwaters (e.g., Benson, Wipfli, Clapcott, & Hughes, 2013; Karlsson et al., 2009). DOC concentrations have a dual role in controlling primary production in food webs, firstly as a carbon source and secondly by determining the photic depth of primary producers (Karlsson et al., 2009). The “shading” effect of DOC has been suggested to be more important than the availability of phosphorous in influencing primary and secondary production in oligotrophic lakes (Karlsson et al., 2009). Increases in DOC in subarctic lakes in both Europe and North American have been predicted with climate change (Zhang et al., 2010), such as a 65% increase to 3.3 mg C/L by 2100 (Larsen, Andersen, & Hessen, 2011). Such increases in DOC are linked to higher runoff from surrounding terrestrial habitats, expanding vegetation cover (and burning) and melting of permafrost releasing carbon into freshwaters (Jaffé et al., 2013; Schädel et al., 2014; Schuur et al., 2008). Combined temperature and nutrient increases in subarctic streams with climate change culminate in increased primary production, as shown from in situ stream experiments in Iceland (Gudmundsdottir, Olafsson, Palsson, Gislason, & Moss, 2011).

Both increasing DOC and terrestrial permafrost thawing contribute to the release and input of phosphorous and nitrogen, leading to productivity and eutrophication in previously oligotrophic subarctic freshwaters (Hobbie et al., 1999; Karlsson et al., 2009). Increased productivity and thermal stratification of deep lakes in northern Alaska since 1975 has led to decreased concentration of hypolimnetic dissolved oxygen (Hobbie et al., 1999). Such effects on oxygen availability have significant implications for the persistence and biotic interactions of aquatic biota, therefore contributing to altered species distribution (e.g., Lindholm, Stordal, Moe, Hessen, & Aass, 2012).

The seasonal timing of organic carbon availability in subarctic freshwaters is expected to be exacerbated by changes in seasonal variation in rain and snowfall. Higher winter snowfall and earlier spring flooding due to warmer temperatures (anticipated with subarctic climate change) contribute to increased nutrient inputs from terrestrial sources (Hinzman et al., 2005). These mechanisms also cause an earlier seasonal initiation of biofilm growth and therefore may alter the temporal dynamics of production and quality of resources in lotic food webs (Davis et al., 2013). For example, the nutrient value of biofilm peaks in spring following an increase in temperature, but decreases during reduced summer discharge (Davis et al., 2013). Furthermore, primary production in subarctic streams is lower under low flow, likely due to the role of water discharge in the transport of nutrients where nutrient availability is low (e.g., Benson et al., 2013). These findings emphasize the role climate plays in determining the timing, quantity, and quality of energy for ecosystem‐level processes.

Despite the substantial body of research examining past or predicted changes in ecosystem productivity associated with climate change in the subarctic, there has been relatively little work to determine the effects on nutritional quality of basal energy sources and the underlying environmental drivers. However, evidence from other regions suggests that effects for basal energy sources will not only be apparent in terms of gross primary production, but also in terms of nutritional quality of energy sources. In an experimental mesocosm study in northern Germany, Verschoor, Van Dijk, Huisman, and Van Donk (2013) reported that elevated dissolved CO_2_ did not affect freshwater algal biomass, but did reduce the nutritional quality of algae. However, it is unclear how the combination of increased gross primary productivity and decreased nutritional quality affects productivity through aquatic food webs, and if effects of increased CO_2_ on nutritional quality are consistent across climates.

### 2.5 Biological invasions

In contrast to changes in the physical and chemical environment due to climate change, ecological consequences of climate change are potentially driven indirectly by the effects of species expanding their distribution beyond their natural range (Gilman, Urban, Tewksbury, Gilchrist, & Holt, 2010; Parmesan, 2006; Wisz et al., 2013). The transition between temperate and subarctic climates is a natural limit for the distribution of many freshwater fish species (e.g., Alofs & Jackson, 2015a; Comte et al., 2013). Increasing temperatures in subarctic regions are consistently associated with poleward range expansions of temperate organisms (Hickling, Roy, Hill, Fox, & Thomas, 2006; Parmesan & Yohe, 2003). Subarctic regions are experiencing widespread poleward invasions of warmer water‐adapted freshwater fish species such as Cyprinids (e.g., ide *Leuciscus idus*, roach *Rutilus rutilus*), Percids (e.g., ruffe*, Gymnocephalus cernua*, European perch, *Perca fluviatilis*), and Centrarchids (e.g., smallmouth bass, *Micropterus dolomieu*, largemouth bass *Micropterus salmoides*) (Alofs & Jackson, 2015a; Chu et al., 2005; Comte et al., 2013; Graham & Harrod, 2009; Sharma, Jackson, Minns, & Shuter, 2007; Van Zuiden, Chen, Stefanoff, Lopez, & Sharma, 2016). As invasive species frequently have strong ecological impacts (Cucherousset & Olden, 2011; Simon & Townsend, 2003), effects of climate change on subarctic freshwaters may be due to indirect mechanisms mediated by poleward invasion of warm‐water‐adapted fish species.

## 3 Ecological Effects of Climate Change on Subarctic Freshwaters Across Different Levels of Organization

### 3.1 Climate change impacts at the individual level

Altered body growth and reproductive performance among individual fish are important impacts of climate change as variation in energy use of ectothermic animals is inherently driven by differences in ambient temperature (Brown, Gillooly, Allen, Savage, & West, 2004; Magnuson, Crowder, & Medvick, 1979). Decreased size and age of sexual maturation with increasing temperature are widely reported for freshwater fish across contrasting climate regions, including populations inhabiting the subarctic (Blanck & Lamouroux, 2007; Daufresne, Lengfellner, & Sommer, 2009; Griffiths, 2006, 2012; Heibo, Magnhagen, & Vøllestad, 2005; Lappalainen, Tarkan, & Harrod, 2008). In contrast to decreasing body size of parental fish, individual egg size increases with increasing temperature in autumn‐spawning brown trout (*Salmo trutta*) and Atlantic salmon (*S*. *salar*; Jonsson & Jonsson, 2009), yet decreases in spring‐spawning roach (Lappalainen et al., 2008). In spring‐spawning European perch, the gonadosomatic index (the proportion of gonad to total body mass) increases with temperature (Heibo et al., 2005), and individual fecundity of roach peaks in mid‐latitude regions throughout their range (Lappalainen et al., 2008). These findings suggest that temperature increases will lead to higher reproductive output for spring‐spawning invasive species (e.g., roach and European perch), potentially enhancing their invasion success in subarctic regions.

Body growth and metabolic rate of individual fish are strongly linked with temperature (Elliott, 1975; Sheridan & Bickford, 2011; Whitney et al., 2016), although influenced by prey availability and ecosystem productivity. However, effects of temperature on body growth rate are inconsistent across the age spectrum of individual fish. For example, during the first 4 years of life of Arctic charr (*Salvelinus alpinus*), temperature has a positive effect on growth only in the second year (Godiksen et al., 2012). Faster juvenile growth of salmonids (e.g., Atlantic salmon, brown trout, whitefish) also occurs with increased temperature, but this effect is dependent on food availability and nutritional value and the increase in temperature relative to optimum temperature for growth (Elliott & Elliott, 2010; Hedger et al., 2013; Kahilainen, Patterson, Sonninen, Harrod, & Kiljunen, 2014). Conversely, increased water temperatures reduce body growth of larger individuals (e.g., Angilletta & Dunham, 2003; Busch, Kirillin, & Mehner, 2012), potentially contributing to expected declines in body size of ectotherms with climate change via increasing metabolic costs (Daufresne et al., 2009; Edeline, La Croix, Delire, Poulet, & Legendre, 2013; Ohlberger, 2013; Sheridan & Bickford, 2011). Importantly, relationships of temperature with body growth and aerobic potential of fish show a hump‐shaped effect indicating an upper threshold response (e.g., Elliott & Hurley, 2000; Whitney et al., 2016). For example, temperature increases of <2.5°C have a positive effect on brown trout growth, but this relationship is inverted for temperature increases of >3°C (Elliott & Elliott, 2010).

Fish body growth rates are influenced by environmental factors beyond temperature and also mediated by effects of climate change at the population, community, and ecosystem levels. In Norwegian streams, for example, brown trout body growth decreases with increasing population density (suggesting prey availability is a primary determinant of growth) (Bærum, Haugen, Kiffney, Moland Olsen, & Vøllestad, 2013). However, increasing water temperature in summer is suggested to increase prey availability, negating the negative relationship between population density and individual growth (Bærum et al., 2013). Body growth of sockeye salmon (*Oncorhynchus nerka*) is linked with the increased production of zooplankton as prey associated with increasing temperature (Schindler, Rogers, Scheuerell, & Abrey, 2005); however, fish body growth decreases with temperature if prey availability is constant (Hill & Magnuson, 1990). In addition, the effects of temperature increases on fish body growth vary depending on food availability (Elliott & Hurley, 2000). For example, in a controlled environment the temperature at which peak energy gain occurs increases when trout were fed increasing proportions of prey fish (Elliott & Hurley, 2000). In subarctic populations of whitefish (*Coregonus lavaretus*), body growth is positively linked with lake productivity (Hayden, et al., 2013b), indicating that the combined effects of temperature, nutrient inputs, and productivity in freshwaters likely cause changes in body growth rates across the life span of fish.

Increased nutrient input from the terrestrial zone to subarctic freshwaters will alter water clarity, therefore impacting the visual‐feeding and foraging performance of subarctic freshwater fish that are predominantly visual feeders requiring high water clarity to see and capture prey (Confer et al., 1978; Kahilainen et al., 2016; Mazur & Beauchamp, 2003). In contrast to cold‐water species (e.g., salmonids), many cool‐ (e.g., percids) and warm‐water species potentially invading subarctic freshwater (e.g., cyprinids) are adapted to foraging in turbid conditions (Confer et al., 1978; Magnuson et al., 1979) and therefore are not disadvantaged by reduced water clarity. Greater prey density with increased water temperature may (partially) counteract effects of decreased water clarity on feeding success (Hayden, et al., 2013b). However, the combined effect of increased water temperature and reduced clarity is predicted to decrease illuminated foraging habitat availability for subarctic fish, thereby reducing body growth (Herb, Johnson, Jacobson, & Stefan, 2014).

Climate change may lead to an intensification of trophic interactions between individuals. Behavior traits such as foraging technique and aggression are related to metabolic activity and growth, both of which are positively correlated with ambient temperature (Lahti, Huuskonen, Laurila, & Piironen, 2002; Nicieza & Metcalfe, 1999). Thus, feeding performance among individuals is influenced by the interaction of temperature, visual conditions, competitor density, ontogeny, and prey availability. As each of these variables is related to a consumers’ environment, individual‐level responses to climate change will likely be complex and relate to each individual's physiological adaptations.

Climate has a strong effect on fish habitat use due to the influence of water temperature and habitat volume. During periods of high water temperatures and low discharge in the main channels of rivers, Atlantic salmon preferentially occupy small groundwater‐fed tributaries as cool habitat refugia (Cunjak, Linnansaari, & Caissie, 2013). Use of thermal refugia by fish also occurs in thermally stratified lakes, where cold‐water species occupy patches with discharge of cold water from groundwater zones (e.g., Snucins & Gunn, 1995) or the colder hypolimnetic zone (Lehtonen, 1996). However, oxygen deficiencies caused by increased nutrient inputs and productivity can restrict the potential for the hypolimnion zone of lakes to function as a thermal refuge for cold‐water species. Such processes may lead to local extirpations if local oxygen concentrations decline beyond the aerobic scope of species (e.g., Herb et al., 2014; Hobbie et al., 1999; Keskinen et al., 2012; Whitney et al., 2016). Loss of thermal refuges may also occur due to effects of strong winds on breakdown of thermal stratification, which is expected in small, shallow lakes (Kernan, Battarbee, & Moss, 2010).

### 3.2 Climate change effects at the population level

Changes in the range distribution of species are the most widely understood effect of climate change as most ectothermic species show a strong unimodal temperature preference (Parmesan & Yohe, 2003). The majority of evidence of range retractions of subarctic fish is based on predicted rather than observed effects (Comte et al., 2013). For example, occurrence rates of walleye, cisco, and Arctic charr are predicted to decline by 22%, 26%, and 73%, respectively, across the subarctic by 2070–2100 in part due to temperature increases under modeled climate scenarios (Hein, Öhlund, & Englund, 2012; Van Zuiden et al., 2016). In conjunction with temperature, range retractions of sensitive cold‐water species, Arctic charr and burbot (*Lota lota*), are also influenced by increases in productivity (Hayden, Myllykangas, Rolls, & Kahilainen, 2017; Jacobson, Stefan, & Pereira, 2010; Lehtonen, 1998; Tammi, Lappalainen, Mannio, Rask, & Vuorenmaa, 1999). Vertical range retractions of cold‐climate species are linked with changes in isotherms causing extinctions at low elevations where water temperature and possibly dissolved oxygen pass physiological performance thresholds (Isaak & Rieman, 2013; Whitney et al., 2016); such reductions in distribution are not consistently compensated for by vertical colonization of higher elevations due to dispersal limitations (e.g., Lindholm et al., 2012). Range expansions and invasion of temperate fish species are also predicted to occur in the subarctic zone due to increased water temperature, further complicating impacts of climate change (described in detail below). For example, smallmouth bass are predicted to increase their range by 55%–422% by 2070 in Ontario, Canada, based on 126 climate scenarios (Van Zuiden et al., 2016). Pike (*Esox lucius*), ruffe, European perch, yellow perch (*Perca flavescens*), smallmouth bass, largemouth bass, roach, and ide have either been observed or predicted to expand their range into subarctic regions due to temperature increases with climate change (Alofs & Jackson, 2015a; Byström et al., 2007; Hayden et al., 2017; Henriksson, Wardle, Trygg, Diehl, & Englund, 2016a; Henriksson, Yu, Wardle, Trygg, & Englund, 2016b; Reist et al., 2006; Sepulveda, Rutz, Ivey, Dunker, & Gross, 2013).

Climate change‐induced temperature increases have implications for the timing and success of reproduction and recruitment (Karjalainen, Keskinen, Pulkkanen, & Marjomäki, 2015; Lappalainen & Tarkan, 2007). Cooling water temperatures during autumn–winter stimulates the spawning of predominantly cold‐water fish species (e.g., salmonids) in subarctic freshwaters (Shuter, Finstad, Helland, Zweimüller, & Hölker, 2012). In contrast, the transition from winter to summer conditions promotes spring spawning of cool‐ and warm‐water species (e.g., cyprinids, percids) (Shuter et al., 2012). Increasing winter temperatures are therefore expected to delay and shorten the spawning period of cold‐water‐adapted subarctic species (putting them at a reproductive disadvantage) and simultaneously stimulate earlier and protracted spawning of cool‐ and warm‐climate species (Karjalainen et al., 2015; Lappalainen & Tarkan, 2007; Shuter et al., 2012).

By altering temperature and hydrological regimes, climate change affects the cohort‐specific survival within freshwater fish populations. Increases in streamflow discharge during early spring contribute to higher mortality of salmon larvae by increasing streambed scouring where eggs are deposited (Cunjak et al., 2013; Goode et al., 2013). Temperature increases stimulate earlier hatching and larval emergence of cool‐water species (e.g., Harper & Peckarsky, 2006). Thermal tolerances for newly hatched salmonids are lower than older juveniles, therefore suggesting that temperature increases will increase larval mortality (Elliott & Elliott, 2010). However, increases in winter temperature by 1–2°C did not alter mortality of eggs and larvae of subarctic salmonids, whitefish, and vendace (Karjalainen et al., 2015), suggesting that extreme events will have the strongest effects on recruitment dynamics. Increased heatwaves will also increase the frequency and extent of fish kills for cohorts of adult or large‐bodied cold‐water salmonids as water temperatures approach the upper lethal limit (Arctic charr and brown trout: 22–25°C; whitefish and Atlantic salmon: 25–28°C; Elliott & Elliott, 2010; Vielma, Koskela, & Ruohonen, 2002). Salmonid (Arctic grayling, Atlantic salmon) population recruitment is also impacted by climate change due to reduced rearing habitat size during low summer discharge, therefore negating positive effects of temperature on individual body growth (Bryant, 2009; Hedger et al., 2013; Hobbie et al., 1999; Jonsson & Jonsson, 2009). In contrast, year‐class strength of cool‐ and warm‐water species (e.g., European perch and roach) is associated with years of warmer temperatures during spawning and early growth (e.g., Jeppesen et al., 2012; Nunn, Frear, Lee, & Cowx, 2010; Tolonen, Lappalainen, & Pulliainen, 2003). Extreme events are unlikely to cause extirpation of fish species in the subarctic and arctic regions (due to the availability of thermal refugia) but, however, may impose significant stress to local populations, such as Arctic charr and burbot, especially in small shallow lakes and streams (Guzzo, Blanchfield, Chapelsky, & Cott, 2016; Lehtonen, 1998). In contrast, prolonged warm summers frequently cause mortality of cisco (*Coregonus artedi*) via oxygen depletion, especially in unstratified lakes in North American lakes (Jacobson, Jones, Rivers, & Pereira, 2008).

### 3.3 Climate change effects at the community level

Climate change alters subarctic freshwater community interactions by influencing behavior and energetic demands of individuals and the abundance and structure of species populations. In turn, biotic interactions influenced by climate can have potential feedbacks for impacts at other levels of organization (e.g., species distribution; Wisz et al., 2013). Across Europe, fish community structure is largely governed by water thermal and productivity regimes and variation occurs as a continuum from cold‐climate northern communities (characterized by low species richness and dominated by salmonids), to cool‐climate (dominated by percid species) and warm‐climate communities in southern regions characterized by high species richness and a dominance on cyprinid fishes (Brucet et al., 2013; Hayden et al., 2017). Populations of cold‐climate species have declined, whereas warmer water species have increased across European lakes associated with climate change during the 20th century (Jeppesen et al., 2012). For example, the range expansion of benthivorous percids (e.g., European perch and ruffe) into subarctic regions due to climate change has increased the prevalence of pelagic feeding by resident whitefish, leading to a lower density and body size of zooplankton, particularly in large and deep lakes (Hayden, Harrod, & Kahilainen, 2014; Hayden, et al., 2013b).

Strong evidence exists that climate change affects biotic interactions within subarctic freshwater communities, leading to changes in community‐level attributes via trophic cascades. Altered abiotic conditions imposed by climate change may reduce the extent of trophic niche partitioning within freshwater systems. For example, whitefish and vendace (*Coregonus albula*) exhibit vertical segregation in deep, cold lakes, thereby reducing resource overlap (Gjelland, Bøhn, & Amundsen, 2007). However, increased temperature and lower dissolved oxygen in the hypolimnion zone linked with climate change is predicted to force both species to occupy the same depth zones likely increasing interspecific competition (Gjelland et al., 2007). Such changes in habitat use by multiple species are associated with increased densities of zooplankton at the warmer surface layers of lakes (due to predation release), yet zooplankton density may decline in the hypolimnetic zone where cold‐water fish become concentrated (Busch et al., 2012). Climate change also alters trophic dynamics via changes in community composition in subarctic lakes. For example, climate change has been linked to increased densities of benthic‐feeding cyprinid species in northern Europe (Comte et al., 2013; Hayden et al., 2017), and their foraging behavior increases nutrient concentrations in the water column leading to higher phytoplankton densities and overall turbidity, which in turn contributes to reduced density of benthic macrophytes (e.g., Volta et al., 2013). However, top‐down control by fish in subarctic freshwaters appears to be dependent on habitat morphology and is more strongly evident in smaller, shallower habitats as opposed to larger and deeper lakes that support complex food webs and contain thermal refugia (Jyväsjärvi et al., 2013). To date, climate change has predominantly contributed to species additions rather than complete replacements. Such additions have led to increased food chain length and food web size in subarctic freshwaters (Thomas et al., 2016; Woodward et al., 2010).

Competitive interactions between fish in subarctic lakes are also driven by the duration of ice cover and timing of ice thaw, both of which are altered by climate change. Reduced body condition of native whitefish and continued active feeding by invasive ruffe are suggestive of increased competition for benthic prey under lake ice, leading to depletion of benthic resources during winter (Hayden, Harrod, Sonninen, & Kahilainen, 2015). Light and temperature limitations during periods of ice cover restrict cladoceran zooplankton population growth almost exclusively to the ice‐free summer months (De Senerpont Domis et al., 2013), which leads to fishes shifting resource use between generalist foraging in summer and benthic foraging in winter (Eloranta, Kahilainen, & Jones, 2010; Hayden, Harrod, & Kahilainen, 2013a). Where brown trout and Arctic charr occur in sympatry, population biomass of brown trout is negatively correlated with ice‐cover duration, suggesting that Arctic charr have a competitive advantage during periods of ice cover (Helland, Finstad, Forseth, Hesthagen, & Ugedal, 2011).

Community‐level responses to climate change in freshwaters are also driven by changes in the timing of recruitment and production of keystone prey or predator species. Temporal changes in the availability of prey and consumer density (i.e., predation pressure) suggest that temporal variation in bottom‐up and top‐down controls of population size within food webs occurs due to the effect of climate (McMeans et al., 2015). For example, lake temperature increases, particularly during spring–summer, lead to higher production and density of zooplankton (e.g., *Daphnia* sp., *Bosmina* sp.) that are important prey sources for fish (Carter & Schindler, 2012). Furthermore, climate change can alter the temporal availability of zooplankton for predators by shortening or extending the period of synchronized predation by multiple consumers (Wagner et al., 2013). For example, *Daphnia* abundances decreased due to increased predation by yellow perch, a cool‐water species which recruited in early summer in lakes during 2004–2010, impacting the growth of late summer hatching bluegill sunfish (*Lepomis macrochirus*) (Kaemingk, Jolley, Willis, & Chipps, 2012).

Fish species invasions mediated by climate change in subarctic lakes have a strong effect on displacing native species, likely by predation. Invasion of northern pike across lakes in Sweden and Alaska has been consistently linked with observed or predicted extirpations of native, cold‐climate adapted subarctic species (Öhlund, Hedström, Norman, Hein, & Englund, 2015; Sepulveda et al., 2013). For example, the establishment of pike as a top predator over 6 years significantly reduced the abundance of native stickleback and eliminated Arctic charr via predation processes in a subarctic lake in Sweden (Byström et al., 2007). Northern pike consumed native salmonids in Alaskan streams, causing significant declines in abundance (Sepulveda et al., 2013). Projected invasions of pike due to climate change are predicted to cause the elimination of brown trout and Arctic charr from 50% and 73%, respectively, of lakes in Sweden by 2100 (Hein, Öhlund, & Englund, 2011, 2014). These changes in species distribution due to community interactions with climate change are dependent on the feeding traits of both native species and the invader. For example, negative associations between invading Centrachid species (e.g., smallmouth bass) and subarctic fish in Ontario, Canada, have been concluded to be more likely driven by consumption rather than competition interactions (Alofs & Jackson, 2015b). However, the generality of this mechanism across subarctic freshwaters is highly dependent on the trophic position of both native and invading species.

### 3.4 Climate change effects at the ecosystem level

Effects of climate change on increased ecosystem productivity in subarctic freshwater will potentially mediate competitive interactions among species and alter physical conditions, contributing to changes at the community and population level. For example, the combined effect of ice‐cover duration and terrestrial energy inputs to freshwater environments are major drivers of climate on interspecific interactions among fish in lakes (Ulvan, Finstad, Ugedal, & Berg, 2012). Specifically, the presence of Arctic charr reduces body growth of brown trout, and these effects are stronger in lakes with high carbon inputs and during periods of ice‐cover than ice‐free summer periods (Ulvan et al., 2012). Increases to productivity in subarctic lakes also reduce the prevalence of marine migrations by anadromous Arctic charr (Finstad & Hein, 2012), potentially contributing to northward changes in their distribution. While eutrophication contributes to increased productivity for food webs (resulting in increased fish biomass), hypereutrophication leads to a decline in dissolved oxygen and the production of toxic and unusable phytoplankton, both contributing to fish kills (Jacobson et al., 2010; Smith, Tilman, & Nekola, 1999) and biodiversity loss (Herb et al., 2014; Jankowski, Schindler, & Lisi, 2014; Tammi et al., 1999). This is especially severe in shallow subarctic lakes, which are covered by ice for 6–8 months each year; increased oxygen demand during this period will greatly increase the likelihood of fish kills. Therefore, the effects of climate change on ecosystem productivity and associated hydrological regimes will be inconsistent due to the terrestrial–aquatic interactions between within these ecosystems.

## 4 Mechanisms Linking Climate Change to Ecological Responses

Ecological effects of environmental change occur via multiple pathways linking different levels of organization. In many circumstances, these effects are first evident at the individual level before becoming evident at population, community, and ecosystem levels (Cucherousset & Olden, 2011; Strayer, 2012). Climate change can impose ecological effects via either individual to ecosystem or ecosystem to individual pathways, which may operate simultaneously (Table 2). Temperature increases in lakes break down niche partitioning between individual fish by forcing competitors into the same depth layers (Busch et al., 2012), increasing predation of zooplankton, and therefore causing top‐down trophic cascades (via individual to ecosystem pathways). In contrast, changes in populations and communities can occur due to increased flux of nutrients and energy from permafrost thaw to aquatic ecosystems, and increased turbidity from terrestrial carbon inputs reduces feeding success of visual‐feeding consumers (Herb et al., 2014), potentially causing localized extirpations due to joint effects of competitive disadvantage and oxygen deficit (Jacobson et al., 2010; Tammi et al., 1999).

**Table 2:** Summary of documented responses and underlying mechanisms by which climate change alters freshwater ecosystems in subarctic regions. Mechanisms identified in brackets indicate that the linkages with other levels of organization. See text for further interpretation

Level	Effect	Mechanism	Sources
Individual	Decreased size and age at sexual maturity	Increased temperature	Blanck and Lamouroux (2007), Daufresne et al. (2009), Griffiths (2006, 2012), Heibo et al. (2005), Lappalainen et al. (2008)
	Altered number and size of eggs (Autumn spawners = increased, Spring spawners = decreased)	Increased temperature	Heibo et al. (2005), Jonsson and Jonsson (2009), Lappalainen et al. (2008)
	Changes to body growth rates across life span	Associated with changes in resource availability (community) that differ with body size, and ecosystem productivity	Angilletta and Dunham (2003), Busch et al. (2012), Elliott and Elliott (2010), Godiksen et al. (2012), Hayden, et al., (2013b), Hedger et al. (2013), Kahilainen et al. (2014)
	Reduced feeding performance for visual‐feeding species	Increased turbidity with increased productivity (ecosystem)	Herb et al. (2014)
	Use of habitats that are within the tolerance limits of individuals within species populations, for example, groundwater‐fed streams and hypolimnion in lakes	Increased temperature, altered hydrology, oxygen deficits	Cunjak et al. (2013), Herb et al. (2014), Hobbie et al. (1999), Kernan et al. (2010), Keskinen et al. (2012), Whitney et al. (2016)
Population	Spawning periods shifted, shortened, eliminated, or extended depending on biological triggers	Increased temperature	Karjalainen et al. (2015), Lappalainen and Tarkan (2007), Shuter et al. (2012)
	Earlier hatching of larvae	Increased spring temperature	Harper and Peckarsky (2006)
	Mortality of larvae	Increased and/ or earlier flooding coinciding with hatching and early growth of larvae	Cunjak et al. (2013), Goode et al. (2013)
	Mortality of juveniles	Increased temperatures during extreme events	Elliott and Elliott (2010), Vielma et al. (2002)
	Altered year‐class strength and cohort size	Environmental and resource conditions (community) in some years conducive of increased juvenile survival and recruitment	Jeppesen et al. (2012), Tolonen et al. (2003)
	Reduced population recruitment in some species	Reduced river flow and habitat size during years of low summer discharge limiting body growth (individual)	Bryant (2009), Hedger et al. (2013), Hobbie et al. (1999), Jonsson and Jonsson (2009)
	Reduced population size	Increased inter‐ and intraspecific (community) competition for resources during summer low water periods	Guzzo et al. (2016), Lehtonen (1998)
	Altered distribution (cold‐climate species reduced, cool or temperate climate expanded or shifted)	Temperature and oxygen regimes shift beyond the critical thresholds where populations can undertake all life‐history processes	Alofs and Jackson (2015a,b), Comte et al. (2013), Isaak and Rieman (2013), Van Zuiden et al. (2016)
Community	Altered community composition	Species‐specific population‐level responses (population)	Brucet et al. (2013), Jeppesen et al. (2012), Hayden et al. (2014, 2017)
	Increased community and food web size	Species invasions occurring faster than species extinctions	Hayden, et al. (2013b), Öhlund et al. (2015), Thomas et al. (2016)
	Increased niche overlap resulting in possible resource competition; top‐down trophic cascade	Multiple species forced to share habitat due to species‐level thermal tolerances (individual)	Byström et al. (2007), Gjelland et al. (2007), Hayden et al. (2015), Hein et al. (2011, 2014), Sepulveda et al. (2013)
	Bottom‐up trophic cascade effects	Temporal mismatching of life‐history dynamics of prey sources and predators (population)	Busch et al. (2012), Carter and Schindler (2012), Kaemingk et al. (2012), Wagner et al. (2013)
Ecosystem	Increased net production, and longer duration of basal energy resources (phytoplankton)	Increased nutrient input from terrestrial zones due to runoff and permafrost thawing	Friberg et al. (2009), Gudmundsdottir et al. (2011), Hinzman et al. (2005), Larsen et al. (2011)
	Lower nutritional quality of primary energy sources to food webs	Increased dissolved CO_2_	Verschoor et al. (2013)
	Altered timing (earlier) of energy inputs by algae and biofilm	Increased temperature; earlier postwinter flooding	Davis, et al. (2013), De Senerpont Domis et al. (2013)
	Reduced nutritional quality of energy sources during seasons of rapid body growth and energy demand	Increased temperature; earlier postwinter flooding	Benson et al. (2013)
	Increased frequency of fish kills	Increased eutrophication and temperature; declines in dissolved oxygen	Jacobson et al. (2010), Smith et al. (1999)

Based on the literature synthesized here, changes in temperature and hydrology appear to be the primary environmental drivers of the ecological response to climate change in subarctic freshwaters (Figure 1). However, both drivers also have indirect secondary effects. For example, thawing of permafrost results in an influx of terrestrial‐sourced nutrients changing productivity regimes (Prowse et al., 2006). Furthermore, while it is often concluded that temperature and hydrological changes are important drivers of climate change impacts, these drivers need to be considered among a broader suite of environmental and ecological drivers (i.e., mechanisms). Without considering the range of mechanisms that affect the structure and functioning of subarctic freshwater ecosystems in reality, the effects of widely studied environmental variables (e.g., temperature) are likely to overstate the importance of such variables. It is clear from studies reviewed here that effects of climate on subarctic freshwaters are variable in space and dependent on habitat and catchment characteristics (e.g., Finstad et al., 2011; Hayden et al., 2014, 2017; Hayden, et al., 2013b). Incorporating additional temporal and spatial covariables into future studies will improve understanding of the mechanisms through which climate change affects freshwater ecosystems and assist identifying environments that function as climate change refugia.

The multitude of ecological mechanisms determining responses to climate, and the variability of those responses identified here for a single specific biome, suggests that there will be some difficulty in determining general principles of ecological responses to climate change at a global scale. Despite apparent consistency of changes in species distributions at regional and global scales across different ecological realms (Comte et al., 2013; Lynch et al., 2016; Scheffers et al., 2016), inconsistencies in responses (i.e., positive, neutral, or negative) within distinct levels of ecological organization in subarctic freshwaters (Table 2) indicate that identifying mechanisms of climate change effects may have limited transferability. This limited transferability is evident across different species (reinforcing the fundamental importance of the biological and ecological traits of taxa in determining how the effects of climate change are manifested) and across different ecological biomes. Such context specificity is not a shortcoming for communicating future biodiversity status and implications for humans due to their unpredictability (e.g., Scheffers et al., 2016). In contrast, understanding the unique effects of climate change for different ecosystem types, biomes, and taxa is necessary for clearer predictive capacity and stronger adaptation potential at regional scales.

## 5 Conceptualizing the Linkages Between Climate Change and Species Invasion Using the Invasion Triangle framework: a case study on Subarctic Freshwaters

Species invasions due to range expansions are a complex aspect of the impact of climate change for ecosystems because the phenomena of invasion are both an effect (response) of climate change and also a potentially important driver (cause) of ecological change (Dukes & Mooney, 1999). The invasion process involves three separate components: the attributes of the invading species, biotic characteristics of the potentially invaded site, and the environmental conditions of the site, collectively termed the “invasion triangle” (Perkins et al., 2011). In addition, once a species establishes following invasion, their subsequent ecological effects may be highly variable as a function of physical and biological factors, but the numbers and characteristics of species in invaded systems could be essential here. Using subarctic freshwater ecosystems as a model, we propose a framework for understanding the factors that influence the process of species invasion due to climate change and the subsequent ecological effects of established invaders (Table 3).

**Table 3:** Interactions between the effects of climate change and climate change‐mediated species invasions hypothesized to occur in subarctic freshwaters

Interaction aspect	Example	References
Climate change alters the vulnerability to be invaded	Heatwaves cause warm‐water temperatures and hypoxia in shallow lakes during summer, leading to widespread mortality of resident cold‐water species (e.g., smelt, vendace), likely facilitating invasion success and competitive advantage of warm‐water species (e.g., roach, bream)	Kangur et al. (2013)
	Reduced density of native, cold‐climate species (Arctic charr) can facilitate the invasion of pike, which in turn produce responses throughout the entire food web	Byström et al. (2007)
	Among lakes, biotic factors such as richness of predators influence invasion vulnerability	Alofs and Jackson (2015a,b)
	Increasing temperature decreases feeding activity of Arctic charr, therefore increasing the competitive advantage of brown trout	Langeland et al. (1991)
Climate change alters potential for species to spread and become invasive	Biological characteristics of invading species match the abiotic conditions of the potentially invaded site	Moyle and Marchetti (2006)
	Increased summer water temperatures increase body growth rates of invaders (e.g., European perch)	Linlokken and Hesthagen (2011)
	Increases in ecosystem productivity with climate change positively affect body growth and population biomass of invading European perch more strongly than increases in temperature	Dubois et al. (2008)
	Abiotic factors, such as growing degree days, determine the regional establishment of smallmouth and largemouth bass	Alofs and Jackson (2015a,b)
Climate change alters the effects of invaders	Increasing turbidity (decreased visibility) reduces visual cues between invasive pike and their prey	Ranåker, Jonsson, Nilsson, and Brönmark (2012)
	Increased recruitment of invaders under invasive conditions leads to higher population densities, which will have stronger competitive effects in invaded ecosystems	Fobert, Fox, Ridgway, and Copp (2011)
	Expansion of invasive species contributes to changes in native species diet, potentially forcing native species to a trophic disadvantage	Corrigan, Winfield, Hoelzel, and Lucas (2011)
	Increasing temperature reduced potential coexistence of invading pike and brown trout, particularly in small lakes	Hein et al. (2014)
	Increasing temperature above 11°C reduced attack rate and speed of invading pike, therefore reducing their ecological effects	Öhlund et al. (2015)
	Invasive brown trout have a stronger effect on communities in warm lakes compared to cold lakes	Finstad et al. (2011)

### 5.1 Effects of climate change on the vulnerability of ecosystems to be invaded

The ecological theory of biological invasion hinges on a paradox between two hypotheses predicting the degree to which native species richness determines an ecosystems vulnerability to invasion (Elton, 1958; Fridley et al., 2007; Jeschke, 2014; Moyle & Light, 1996). First, the diversity‐invasibility hypothesis, proposed by Elton (1958), suggests that ecosystems are more vulnerable to invasion when they have low species richness as invading populations face little resource competition or predation during establishment (e.g., Eisenhauer, Schulz, Scheu, & Jousset, 2013). The second, alternative, hypothesis is that increasing native species richness enhances invasion opportunities due to greater habitat heterogeneity and resources, and reflected by a positive relationship between native and invasive species richness (e.g., Fridley et al., 2007; Stohlgren, Barnett, & Kartesz, 2003).

Under both scenarios, climate change can be hypothesized to increase the vulnerability of ecosystems to invasion in subarctic regions. Environmental disturbances deplete the abundance or richness of organisms in ecosystems, therefore increasing the vulnerability of ecosystems to invasion by creating vacant (or under‐exploited) niches available to be filled by new species (Donohue et al., 2013; Moyle & Light, 1996; Von Holle, 2013). The diversity‐invasibility hypothesis is relevant for subarctic freshwaters because they naturally support communities of relatively few, often generalist, species (Eloranta et al., 2015; Henriksson, et al., 2016b; Vincent & Laybourn‐Parry, 2008) that are strongly influenced by climate. For example, extreme heatwaves causing hypoxia and increased water temperatures in shallow lakes during summer may lead to widespread mortality of resident cold‐water species (Arctic charr, burbot, vendace, cisco, smelt *Osmerus eperlanus*) and facilitating the invasion success and competitive advantage of cool‐ or warm‐water species (e.g., ruffe) (Jacobson et al., 2010; Kangur et al., 2013; Keskinen et al., 2012; Lehtonen, 1998). Increasing water temperatures reduce the feeding activity of individual Arctic charr, therefore facilitating the invasion success and establishment of northern pike or community dominance of brown trout (Byström et al., 2007; Finstad et al., 2011; Langeland, L'Abeelund, Jonsson, & Jonsson, 1991). Changes in ecosystem productivity also influences competitive dominance and biological resistance to invasion in subarctic lakes. For example, Arctic charr were better able to exploit prey resources than brown trout in cold, low‐productivity lakes when compared to warm productive lakes (Finstad et al., 2011). In relation to the second hypothesis (positive native‐exotic species richness), climate change is increasing regional species richness in subarctic regions (Harrod, 2015; Hayden, Harrod, & Kahilainen, 2013a; Hayden, Holopainen, et al., 2013b, 2013b). As subarctic freshwaters become environmentally suitable for cool‐ and warm‐water‐adapted species, the increase in species richness is unlikely to represent a deterrent to further invasion, potentially resulting in an “invasion meltdown” scenario (where the invasion of one species facilitates others and enhances effects for native ecosystems; Simberloff & Von Holle, 1999). Furthermore, by definition much of this increase in species richness will be a consequence of invasion by nonresident cool‐ and warm‐water‐adapted species.

### 5.2 Effects of climate change on the potential for species to become invasive

Climate change affects the potential distribution of species and these influences are best known in terms of range expansions of species beyond their natural range (Comte et al., 2013; Parmesan, 2006). Such range expansions occur because climate change alters the physical environmental characteristics of ecosystems, and when these characteristics match those required by a species to occupy and function, that species has the potential to invade and persist in that ecosystem (Moyle & Marchetti, 2006). Species with broad environmental tolerances and plasticity of growth across the life span exhibit high potential to invade previously unoccupied habitats (Britton, Cucherousset, Davies, Godard, & Copp, 2010; Budy et al., 2013; Hayden et al., 2014; but see Sax et al., 2007). Across reported predictions of the global effect of climate change on freshwater fish, only warm‐water taxa are expected to show range expansions (Comte et al., 2013), but field evidence from subarctic Europe suggests that both cool‐ and warm‐water species range is expanding (Hayden et al., 2014, 2017). However, an important caveat to the impacts of invasion is that biological performance of invasive species is not consistent when compared across native and invaded populations (Rypel, 2014), therefore contributing to unsuccessful establishment (Zenni & Nuñez, 2013).

Climate change is predicted to increase the potential for species to invade and expand their distribution northward into subarctic regions of North America, Europe, and Asia (e.g., Alofs & Jackson, 2015a; Prowse et al., 2006). The northern distribution of smallmouth bass is restricted by minimum temperature variables; therefore, climate change may be expected to increase the potential for northward invasion into Canada (Jackson & Mandrak, 2002; Sharma et al., 2007). Sportfish, including both smallmouth and largemouth bass, expanded their range northward by 17.9 km per decade across Ontario, Canada (Alofs, Jackson, & Lester, 2014), and these expansions are limited primarily by abiotic factors related to climate at the regional scale (Alofs & Jackson, 2015a). Body growth rate and population abundance of European perch are positively driven by temperature and ecosystem productivity (Dubois, Gillet, Hilgert, & Balvay, 2008; Linlokken & Hesthagen, 2011); increases in these variables with climate change will enhance the further success of subarctic invasion by European perch (Hayden et al., 2014).

### 5.3 Influence of climate on the ecological effects of invasive species

The impacts of invasive species are well understood, particularly for non‐native freshwater fish introduced via human‐assisted dispersal, and these impacts are apparent across the full spectrum of ecological organization (e.g., Cucherousset & Olden, 2011; Gallardo, Clavero, Sanchez, & Vila, 2016; Simon & Townsend, 2003; Thomas et al., 2016). However, there remains a dearth of empirical evidence to determine whether the impacts of climate change‐mediated invasions are consistent following climate related modification of invaded habitats (Rahel & Olden, 2008). Specifically, are the impacts of invasive species consistent with climate change, or under what situations are the effects of climate change and invasive species synergistic or antagonistic? These considerations are of both ecological and economic importance. For example, if climate change reduces the ecological effects of an invasive species, then the performance of conservation management actions targeting invasive species could be predicted to be less effective when compared to another invader whose effects are synergistic under climate change. We therefore emphasize that the ecological effects of invasive species are considered under the context of different climate change scenarios, and this entails more than simply predicting future distributions of invaders.

Ecological effects of species invasions vary due to the direct environmental and ecological effects of climate change. This suggests that some invasive species will have more benign ecological effects under future climate scenarios when compared to past or present conditions. For example, northern pike and European perch are two species expanding their range within subarctic regions (Hayden et al., 2014; Hein et al., 2012). Increased temperature coupled with invasion of northern pie is expected to cause the extirpation of both native brown trout and Arctic charr, particularly in small lakes (Byström et al., 2007; Hein et al., 2014; Öhlund et al., 2015). This synergistic effect may be considered a consequence of stronger recruitment of invaders in newly invaded habitats leading to higher population densities, and potentially to stronger competitive interactions. This mechanism would be exacerbated by climate change modifying the habitat to resemble the invaders native range. The growth efficiency of Arctic charr is double that of brown trout in cold, low‐productivity lakes, whereas brown trout are more competitively aggressive in warm, productive lakes, suggesting that competitive interactions between invasive and native species along a gradient of productivity determine the effects of invaders (Finstad et al., 2011). Ecological impacts of invasive species on native species are positively related with temperature in more temperate systems (Habit et al., 2010), suggesting that initial impacts of range‐expanding species may be limited and become stronger over time with increasing temperature and growing population sizes.

## 6 Conclusions

This synthesis highlights the complex interplay between multiple environmental variables and invasive species in determining the effects of climate change in subarctic freshwater ecosystems across multiple levels of organization. Explicit integration of the effects of climate change and invasive species will produce more robust predictions and improved understanding of underlying mechanisms of the each stressor at both regional (e.g., subarctic) and global spatial extents. However, despite the concern of climate change and the subsequent risks to ecosystems, biodiversity and ecosystem services, this synthesis also highlights three key knowledge gaps relevant to drawing inferences and making predictions of the effects of climate change on both subarctic freshwaters and other ecosystem types. These gaps limit our understanding of the mechanisms by which of climate change induces ecological effects, which in turn restricts the ability to make strong predictions of climate change impacts.

1. *What is the role of habitat characteristics (e.g., size, depth, disturbance regime) and landscape (e.g., connectivity) context in the effects of climate change?* In freshwater systems, we have a poor understanding of how ecological effects of climate are mediated by habitat characteristics. Much of this is due to limited replication of studies (sensu Belovsky et al., 2004) across landscapes, species, and habitats. However, there is evidence to suggest that the effects of climate on freshwaters vary on the basis of habitat characteristics (lotic vs. lentic, habitat morphology) and landscape context, and knowledge of these interactions will be useful in future predictive modeling.
2. *What are the combined ecological effects of climate change and non‐native invasive species in freshwater ecosystems?* Inadequate consideration of the role of climate in mediating the effects of non‐native species (where invasion is facilitated by climate change) means there is a risk in extrapolating knowledge of invasive species impacts as being consistent under different climate scenarios or along strong climate gradients. This question is important to identify the extent to which non‐native species are drivers, passengers, or “backseat drivers” (sensu MacDougall & Turkington, 2005; Bauer, 2012) of ecological impacts of climate, which is critical for future management options. Because dispersal and invasion of freshwater fish are constrained by hydrological connectivity, freshwater ecosystems are highly relevant to tease apart the effects of these stressors by carefully designed surveys along climate gradients across hydrological networks that vary in the degree to which invasion is possible (due to dispersal barriers constraining range expansions). Thomaz et al. (2012) determined that the combined use of space‐for‐time and time approaches improves the accuracy of conclusions of the impacts of non‐native species. The use of natural gradients in variables that are expected to change with climate change continues to be an effective basis for predicting future climate change consequences and identify underlying ecological mechanisms (Fukami & Wardle, 2005; Woodward et al., 2010).
3. *How do the mechanisms linking climate change with ecological responses determine patterns of freshwater biodiversity across local, landscape, and regional spatial scales?* An extensive body of evidence details how freshwater biodiversity in northern latitude regions is changing in association with climate change (Buisson et al., 2013; Comte et al., 2013; Heino et al., 2009; Sharma et al., 2007). However, the mechanisms through which these changes are occurring are relatively less well known. Subarctic freshwaters are ideal systems to study how changes in biodiversity under current and future climate change will influence trophic interactions, food web structure, ecosystem function, and subsequent consequences for humans.

## Conflict of Interest

None declared.
